# Determinants of exclusive breastfeeding for the first six months in China: a cross-sectional study

**DOI:** 10.1186/s13006-021-00388-y

**Published:** 2021-05-17

**Authors:** Huifeng Shi, Yumei Yang, Xiaohan Yin, Jia Li, Jin Fang, Xiaoli Wang

**Affiliations:** 1 Department of Obstetrics and Gynecology, Peking University Third Hospital, Beijing, China; 2 Department of Maternal and Child Health, Peking University School of Public Health, Beijing, China; 3 School of Economics and Management, Beijing Forestry University, Beijing, China; 4 China Development Research Foundation, Beijing, China; 5 National Health Commission Key Laboratory of Reproductive Health, Beijing, China

**Keywords:** Exclusive breastfeeding, Determinants, China

## Abstract

**Background:**

Breast milk is the best source of essential nutrients and bioactive components for infants under 6 months. However, little is known about what affects breastfeeding intentions and practices of Chinese mothers. With measures of individual, setting, and sociocultural factors, this study examined determinants of exclusive breastfeeding in the first 6 months of infancy in China.

**Methods:**

Data were obtained from a national cross-sectional survey in China in 2018 that included 5237 infants under 6 months with available measurements of breastfeeding. A 24-h reported food recall method was applied to assess breastfeeding and complementary food intake in the past 24 h. Potential breastfeeding determinants categorized into six aspects were measured: (1) infant health, (2) maternal sociodemographic characteristics, (3) maternal health, (4) breastfeeding support from family, friends, and workplace, (5) social support for breastfeeding, and (6) maternal breastfeeding experiences and knowledge. Reasons for non-commencement or early cessation of breastfeeding were evaluated for non-breastfed infants. For breastfed infants, multivariate logistic regression was used to explore the determinants of exclusive breastfeeding.

**Results:**

About 30 % (29.5%) of infants under 6 months were exclusively breastfed; 2.3% (2.3%) had never been breastfed and 3.2% had ceased breastfeeding. No breast milk (60.7%), maternal illness (13.9%), and infant illness (13.1%) were the top three reasons for non-commencement of breastfeeding. Insufficient breast milk was the reason given for ceasing breastfeeding early by almost two thirds of caregivers who had stopped breastfeeding. The following factors were associated with exclusive breastfeeding: maternal higher education, formal employment with ≥6 months of paid maternity leave, support of the husband and best friends for breastfeeding, a breastfeeding-supportive society, and better breastfeeding knowledge and experiences (a previous successful breastfeeding experience ≥6 months and early initiation of breastfeeding). Maternal age of ≥40 years, caesarean delivery, and infant disease history were associated with non-exclusive breastfeeding.

**Conclusions:**

The exclusive breastfeeding rate is still very low in China. Multidimensional barriers contribute to this situation. A comprehensive intervention framework is needed to increase optimal breastfeeding and achieve substantial public health gains.

## Background

Breast milk is the best source of essential nutrients and bioactive components for infants for the majority of time [1, 2]. Strong evidence supports that breastfeeding is an important and even the most cost-effective intervention against infant obesity, diabetes, infections, cardiovascular disease, developmental delay, and deaths, as well as maternal breast cancer and diabetes [3, 4]. Exclusive breastfeeding for the first 6 months with continued breastfeeding for up to 2 years of age is recommended by many health institutions such as the World Health Organization (WHO) [5]. The 2012 World Health Assembly (WHA) set one of the global nutrition target as increasing the exclusive breastfeeding rates up to 50% by 2025 [6]. According to estimates, the exclusive breastfeeding rate measured by a 24-h diet recall increased from 24.9% in 1993 to 35.7% in 2013 globally; the corresponding rates were 47% in low-income countries, 39% in lower-middle-income countries, and 37% in upper-middle-income countries [4]. In China, as estimated by a nationally representative survey in 2013, 20.7% of infants under 6 months were exclusively breastfed according to the reported diets during the 24 h [7]. The Under-5 Child Nutrition and Health Surveillance System shows that the prevalence of exclusive breastfeeding measured by diet recall of the previous 24 h increased from 16.14% in 2013 to 34.90% in 2018 in China, with an annual percent of change of 14.90% [8].

A wide range of factors may impede breastfeeding. Some maternal sociodemographic characteristics and health problems were identified to be associated with early breastfeeding cessation, including low maternal education [9, 10], pre-pregnancy overweight/obesity [11], caesarean delivery [9], and maternal health problems [12]. Previous findings showed disparities in the early introduction of complementary foods according to social and cultural context [13–17]. However, we know little about what affects breastfeeding intentions of Chinese mothers, especially the effects of setting (e.g. family, peers, hospital, and workplace) and sociocultural factors because too little research was done. Furthermore, different set of determinants of exclusive breastfeeding may be involved at different months of age. More comprehensive observation of the breastfeeding determinants is needed. The Lancet Breastfeeding Series Group has proposed a conceptual model for the components of an enabling environment for breastfeeding [18]. In the conceptual model, the determinants affecting breastfeeding decisions and behaviors was classified into three levels: (1) individual determinants, including mother and infant health and attributes, and mother–infant relationship; (2) setting determinants, including health systems and services, family, community and workplace environment for breastfeeding, and employment status; (3) structural determinants, including social and cultural attitudes and market factors for breastfeeding [18]. This framework provides a theoretical basis for exploring the determinants of breastfeeding in different sociocultural context.

Using data from a recent cross-sectional survey, this study aimed to explore determinants associated with maternal practices of exclusive breastfeeding in China. Based on previous findings, we systematically measured multi-dimensional potential factors of breastfeeding in sociocultural, setting, and individual levels. It is hoped that this study will contribute to our increased understanding of exclusive breastfeeding practices and development of more effective breastfeeding interventions.

## Methods

### Areas and subjects

A cross-sectional survey on breastfeeding was conducted among infants under 12 months of age in China in 2018. Multi-stage stratified cluster random sampling was used. First, all county-level administrative units (counties and county-level cities/districts) of 31 provinces, autonomous regions and municipalities in mainland China were categorized into four strata: large cities (135 central districts of municipalities, cities with separate plans and provincial capital cities with more than 1 million urban residents), middle and small cities (1086 county-level cities/districts and non-central districts of the large cities), general rural areas (1074 non-poverty-stricken counties) and poor rural areas including 559 poverty-stricken counties identified in the Outline for Development-oriented Poverty Reduction for China’s Rural Areas (2011–2020), except for county-level cities/ districts categorized as middle and small cities. Then, four large cities, four middle and small cities, two general rural areas, and two poor rural areas were selected with Probability-Proportional-to-Size sampling method according to the number of under-12-month-old infants in the Expanded Program on Immunization in 2014, but the selected rural areas were replaced by other areas at the same stratum given the implication of the following intervention program. Second, four communities/ townships were randomly selected by the probability of population proportion in each selected county-level unit. Finally, about 18 infants from each month age group were randomly sampled from the list of infants in the Expanded Program on Immunization in each selected community or township. In selected cities, household registered children and migrant children whose mothers migrated from other counties for at least 1 month were sampled in the same proportion.

Infants under 12 months whose mothers or primary caregivers agreed to participation, had no mental illness and could clearly answer the questions were enrolled. The caregivers were face-to-face interviewed about feeding practices and potential determinants. All aspects of the study were approved by the Ethics Review Board of the National Institute for Nutrition and Health, Chinese Center for Disease Control and Prevention (No. 2016–015), and written informed consent was obtained from caregivers before the interview. Finally, a total of 10,408 infants under 12 months were enrolled in the original survey. According to the research objectives, our analysis only used the data of 5237 infants under 6 months with available breastfeeding measurements (50 were excluded with missing data).

### Measurements

Infant breastfeeding practices and the potential factors were measured using face-to-face interviews with the caregivers by uniformly trained investigators. Selection of these factors were based on the conceptual model for breastfeeding determinants and interventions proposed by the Lancet Breastfeeding Series Group [18].

#### Breastfeeding indicators

A 24-h reported food recall method was applied to assess breastfeeding and complementary food intake in the past 24 h, as recommended in *Indicators for Assessing Infant and Young Child Feeding Practices* by WHO [19]. Exclusive breastfeeding was defined as feeding infants under 6 months exclusively with breast milk during the previous day. Predominant breastfeeding was identified when infants under 6 months received breastmilk as the predominant source of nourishment and did not fed with semi-solid / solid foods or other liquids except for oral rehydration solution, vitamin and/or mineral supplements, ritual fluids, water and water-based drinks, and fruit juice during the previous day. Early breastfeeding cessation was identified when an infant had been breastfed ever but already weaned at the time of investigation.

#### Measurements of potential determinants

Resident areas, sex and month age of infants were recorded. Potential breastfeeding determinants categorized into six aspects were measured: (1) infant health: delivery mode, preterm birth, and disease history; (2) maternal sociodemographic characteristics: age, ethnic origin, and education level; (3) maternal health: pre-pregnancy height, weight, complications during pregnancy and parturition; (4) setting support: whether family members and the best friends of mothers supported for breastfeeding, whether health institutions provided breastfeeding education, and whether workplace provided paid maternity leave; (5) social support: mothers were asked whether they fed their child with formula or reduced going out because of discomfort when breastfeeding in public places, and whether she was embarrassed about public breastfeeding; then social support for breastfeeding was defined as the answer to all these questions was no; and (6) maternal breastfeeding experiences and knowledge: we also interviewed maternal breastfeeding experience, early initiation of breastfeeding, and understanding of the perception of exclusive breastfeeding and the benefits of breastfeeding (measured with a 11-item questionnaire). For non-breastfed children, the reasons for never being breastfed or early breastfeeding cessation were interviewed using multiple-choice questions.

### Statistical analysis

The proportions of different feeding practices (never being breastfed, early breastfeeding cessation, exclusive breastfeeding, and non-exclusive breastfeeding) were calculated and their change over the first 6 months of infancy was presented by a figure.

For non-breastfed infants, the proportions of reasons for never being breastfed or early breastfeeding cessation were calculated. For breastfed infants, the association of the interested factors with exclusive breastfeeding was presented using adjusted odds ratios (aORs) with 95% confidence intervals (CIs) by employing the multivariate logistic regression that adjusted for resident areas, infant sex and age of months, and other factors of the first five aspects aforementioned. The multivariate logistic regression was also performed to examine the association of maternal breastfeeding experiences and knowledge with exclusive breastfeeding among infants being breastfed, adjusting for resident areas, infant sex and age of months, and factors identified by the previous model to be associated with exclusive breastfeeding. In the subgroup analysis, we divided breastfed infants under 6 months into three groups (0–1, 2–3, and 4–5 months) according to their age, and similar multivariable analyses were conducted as described above in each group to identify potential determinants for exclusive breastfeeding at different age of months. In these multivariable analyses, we used listwise deletion for variables with < 1% missing data, and assigned a category “unknown” to the missing value of categorial variables with ≥1% missing data instead of excluding these cases.

All of the analyses were performed using Statistical Package for the Social Sciences (SPSS) software 20.0 (SPSS, Inc., Chicago, IL). Two-sided *p* values of less than 0.05 were deemed to be statistically significant.

## Results

Among 5237 infants included, around two thirds of them were in cities, 50.0% were boys and those in each month age group accounted for 15.6 –18.2% of all surveyed infants (Table 1). 86.1% of the mothers were of Han ethnic group and 13.9% were of ethnic minorities. 15.5% of the mothers were 35 years old or older and 39.4% had a college or higher school education.

**Table 1:** Socio-demographic characteristics of surveyed infants under 6 months, *n* (%)

Characteristics	0–1 months	2–3 months	4–5 months	Total
	(*n* = 1770)	(*n* = 1750)	(*n* = 1717)	(*n* = 5237)
Areas				
Large cities	632 (35.7)	617 (35.3)	593 (34.5)	1842 (35.2)
Middle and small cities	586 (33.1)	569 (32.5)	550 (32.0)	1705 (32.6)
General rural areas	270 (15.3)	281 (16.1)	288 (16.8)	839 (16.0)
Poor rural areas	282 (15.9)	283 (16.2)	286 (16.7)	851 (16.2)
Child sex				
Boys	890 (50.3)	883 (50.5)	845 (49.2)	2618 (50.0)
Girls	880 (49.7)	867 (49.5)	872 (50.8)	2619 (50.0)

### Exclusive breastfeeding status

**Fig. 1:**
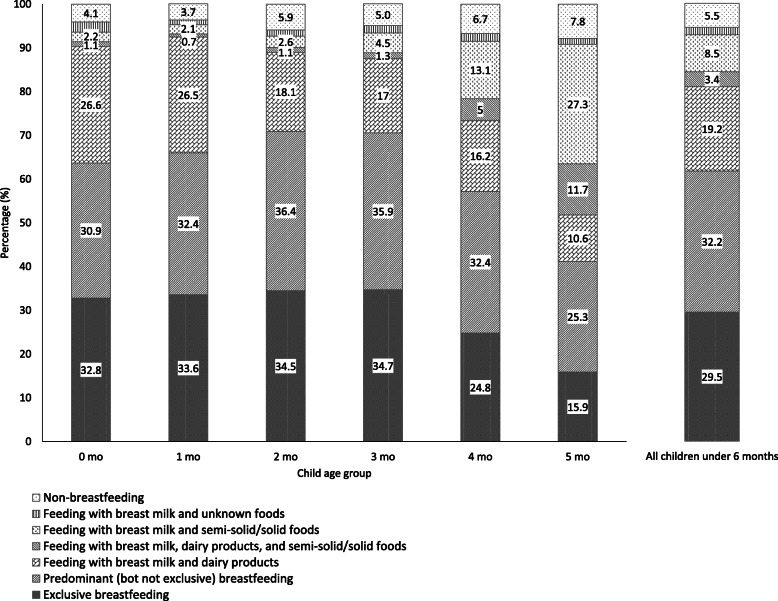
Feeding practices in the first six months of infancy (*N* = 5237)

Of 5237 infants under 6 months of age and included in the analyses, 94.5% were breastfed. The overall exclusive breastfeeding rate was 29.5%; it ranged from 32.8 to 34.7% among infants in the first 4 months, but was lower among 4-month and 5-month infants (24.8 and 15.9% respectively). The non-breastfeeding rate ranged from 4.1 to 5.9% among infants in the first 4 months, and was 6.7% among 4-month infants and 7.8% among 5-month infants. For more details on breastfeeding practices see Fig. 1.

### Reasons for non-commencement and early cessation of breastfeeding

**Fig. 2:**
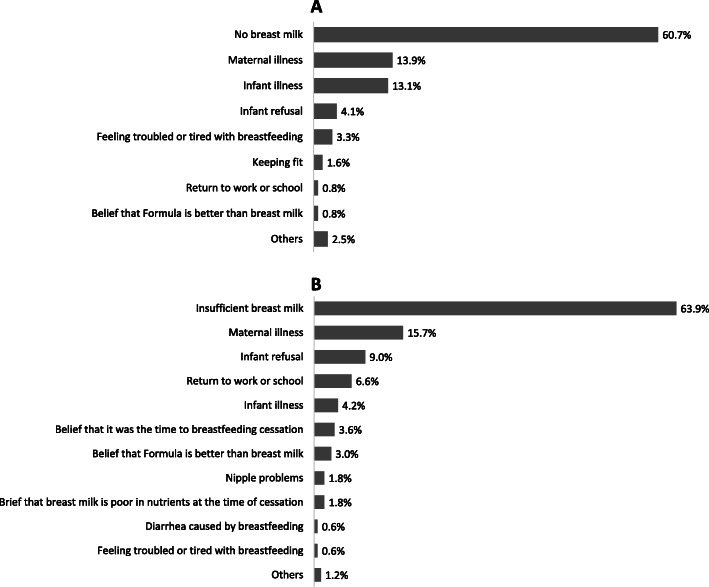
Reasons for non-commencement (*n* = 122) and early cessation (*n* = 166) of breastfeeding among surveyed children under six months. **a**. Reasons for non-commencement of breastfeeding; **b**. Reasons for early breastfeeding cessation.

One hundred and twenty-two (2.3%) infants had never been breastfed. One hundred and sixty-six (3.2%) infants were ever breastfed but had been ceased at the survey time. The top five reasons, included no breast milk, maternal illness, infant illness, infant refusal, and feeling troubled or tired with breastfeeding, and accounted for 60.7, 13.9, 13.1, 4.1, and 3.3% of all infants never being breastfed, respectively (Fig. 2a). The top five reasons for early breastfeeding cessation were perception of insufficient breast milk (63.9%), maternal illness (15.7%), infant refusal (9.0%), return to work or school (6.6%), and infant illness (4.2%) (Fig. 2b).

### Determinants of exclusive breastfeeding among breastfed infants under six months

According to the results of multivariable adjusted analyses, higher exclusive breastfeeding proportions were found among breastfed infants under 6 months with the following characteristics, compared to the counterparts: (1) maternal education level of college school or higher (aOR 1.32; 95% CI 1.08, 1.61); (2) support for breastfeeding from the husbands (aOR 1.72; 95% CI 1.18, 2.51) and best friends (aOR 1.64; 95% CI 1.19, 2.26); (3) mothers being formally employed with ≥6 months of paid maternity leave (aOR 2.77; 95% CI 1.65, 4.65), and (4) social support for breastfeeding (aOR 1.49; 95% CI 1.24, 1.78). Caesarean delivery (aOR 0.80; 95% CI 0.70, 0.92), infant disease history (aOR 0.71; 95% CI 0.63, 0.81), and maternal age of ≥40 years (aOR 0.56; 95% CI 0.35, 0.91) were associated with lower probability of exclusive breastfeeding among breasted infants (Table 2).

**Table 2:** Determinants of exclusive breastfeeding for breastfed infants under 6 months

	0–1 months		2–3 months		4–5 months		Total	
	EBF/BF (%)	aOR (95%CI) (*n* = 1667)	EBF/BF (%)	aOR (95%CI) (*n* = 1628)	EBF/BF (%)	aOR (95%CI) (*n* = 1562)	EBF/BF (%)	aOR (95%CI) (*n* = 4857)
**Child health**								
Caesarean delivery ^a^								
No	396/1087 (36.4)	1.00 (reference)	389/1001 (38.9)	1.00 (reference)	230/982 (23.4)	1.00 (reference)	1015/3070 (33.1)	1.00 (reference)
Yes	190/613 (31.0)^*^	0.89 (0.70, 1.12)	215/651 (33.0)^*^	**0.77 (0.62, 0.97)**	121/608 (19.9)	**0.72 (0.55, 0.94)**	526/1872 (28.1)^*^	**0.80 (0.70, 0.92)**
Preterm ^b^								
No	573/1637 (35.0)	1.00 (reference)	583/1582 (36.9)	1.00 (reference)	333/1519 (21.9)	1.00 (reference)	1489/4738 (31.4)	1.00 (reference)
Yes	10/52 (19.2)^*^	0.49 (0.24, 1.03)	15/52 (28.8)	0.81 (0.43, 1.52)	16/57 (28.1)	1.17 (0.62, 2.22)	41/161 (25.5)	0.78 (0.53, 1.13)
Disease history ^a^								
No	411/1026 (40.1)	1.00 (reference)	312/761 (41.0)	1.00 (reference)	146/691 (21.1)	1.00 (reference)	869/2478 (35.1)	1.00 (reference)
Yes	172/660 (26.1)^*^	**0.56 (0.45, 0.71)**	290/885 (32.8)^*^	**0.68 (0.55, 0.84)**	200/887 (22.5)	1.05 (0.81, 1.36)	662/2432 (27.2)^*^	**0.71 (0.63, 0.81)**
**Maternal sociodemographic characteristics**								
Age (years) ^a^								
< 25	113/327 (34.6)	1.00 (reference)	124/318 (39.0)	1.00 (reference)	60/315 (19.0)	1.00 (reference)	297/960 (30.9)	1.00 (reference)
25–29	244/675 (36.1)	1.07 (0.79, 1.44)	231/655 (35.3)	**0.65 (0.48, 0.88)**	127/592 (21.5)	0.90 (0.62, 1.30)	602/1922 (31.3)	0.86 (0.72, 1.04)
30–34	147/443 (33.2)	0.91 (0.64, 1.27)	156/424 (36.8)	**0.70 (0.50, 0.99)**	104/423 (24.6)	1.09 (0.73, 1.63)	407/1290 (31.6)	0.87 (0.71, 1.06)
35–39	76/225 (33.8)	0.89 (0.59, 1.33)	81/213 (38.0)	0.70 (0.47, 1.05)	52/207 (25.1)	1.05 (0.66, 1.68)	209/645 (32.4)	0.87 (0.68, 1.11)
40-	6/26 (23.1)	0.52 (0.19, 1.41)	12/40 (30.0)	0.49 (0.23, 1.04)	8/51 (15.7)	0.54 (0.23, 1.30)	26/117 (22.2)	**0.56 (0.35, 0.91)**
Ethnic origin ^a^								
Han	495/1479 (33.5)	1.00 (reference)	533/1411 (37.8)	1.00 (reference)	308/1353 (22.8)	1.00 (reference)	1336/4243 (31.5)	1.00 (reference)
Other	90/217 (41.5)^*^	**1.41 (1.00, 1.98)**	73/241 (30.3)^*^	**0.65 (0.47, 0.92)**	43/237 (18.1)	0.83 (0.55, 1.27)	206/695 (29.6)	0.93 (0.76, 1.13)
Education level ^a^								
Middle school or below	217/694 (31.3)	1.00 (reference)	218/697 (31.3)	1.00 (reference)	126/693 (18.2)	1.00 (reference)	561/2084 (26.9)	1.00 (reference)
High school	109/326 (33.4)	1.18 (0.87, 1.62)	107/301 (35.5)	1.15 (0.83, 1.58)	56/281 (19.9)	1.03 (0.70, 1.52)	272/908 (30.0)	1.15 (0.95, 1.39)
College school or higher	258/675 (38.2)^*^	**1.44 (1.03, 2.01)**	279/653 (42.7)^*^	1.24 (0.89, 1.74)	169/615 (27.5)^*^	1.19 (0.81, 1.75)	706/1943 (36.3)^*^	**1.32 (1.08, 1.61)**
**Maternal health**								
Height (cm) ^b^								
First Quarter	122/373 (32.7)	1.00 (reference)	150/402 (37.3)	1.00 (reference)	82/393 (20.9)	1.00 (reference)	354/1168 (30.3)	1.00 (reference)
Second Quarter	194/584 (33.2)	0.93 (0.69, 1.25)	189/577 (32.8)	0.77 (0.57, 1.02)	124/570 (21.8)	0.96 (0.68, 1.35)	507/1731 (29.3)	0.86 (0.73, 1.03)
Third Quarter	98/277 (35.4)	1.02 (0.71, 1.46)	91/263 (34.6)	0.81 (0.57, 1.15)	55/219 (25.1)	1.21 (0.79, 1.85)	244/759 (32.1)	0.94 (0.76, 1.16)
Fourth Quarter	137/402 (34.1)	0.93 (0.67, 1.29)	144/357 (40.3)	0.93 (0.67, 1.29)	69/346 (19.9)	0.77 (0.52, 1.15)	350/1105 (31.7)^*^	0.89 (0.73, 1.08)
Body-mass index ^b^								
Normal weight (18.5–23.9 kg/m^2^)	363/1019 (35.6)	1.00 (reference)	371/1013 (36.6)	1.00 (reference)	216/981 (22.0)	1.00 (reference)	950/3013 (31.5)	1.00 (reference)
Underweight (< 18.5 kg/m^2^)	74/206 (35.9)	1.06 (0.76, 1.49)	71/211 (33.6)	0.90 (0.64, 1.26)	33/183 (18.0)	0.76 (0.49, 1.18)	178/600 (29.7)	0.94 (0.76, 1.15)
Overweight (24.0–27.9 kg/m^2^)	74/285 (26.0)	**0.68 (0.50, 0.93)**	92/269 (34.2)	1.06 (0.78, 1.43)	57/249 (22.9)	1.06 (0.74, 1.53)	223/803 (27.8)	0.90 (0.75, 1.08)
Obesity (≥28.0 kg/m^2^)	26/98 (26.5)^*^	0.67 (0.41, 1.09)	34/92 (37.0)	1.17 (0.73, 1.86)	23/104 (22.1)	1.03 (0.61, 1.72)	83/294 (28.2)^*^	0.93 (0.70, 1.23)
Complications during pregnancy or parturition ^b^								
No	531/1511 (35.1)	1.00 (reference)	531/1446 (36.7)	1.00 (reference)	306/1396 (21.9)	1.00 (reference)	1368/4353 (31.4)	1.00 (reference)
Yes	50/171 (29.2)	0.74 (0.50, 1.08)	67/186 (36.0)	0.92 (0.65, 1.30)	41/174 (23.6)	0.94 (0.62, 1.40)	158/531 (29.8)	0.87 (0.70, 1.07)
**Setting and social support for breastfeeding**								
Support from husbands ^b^								
No	27/130 (20.8)	1.00 (reference)	35/128 (27.3)	1.00 (reference)	19/116 (16.4)	1.00 (reference)	81/374 (21.7)	1.00 (reference)
Yes	546/1544 (35.4)^*^	1.40 (0.72, 2.71)	564/1509 (37.4)^*^	**1.91 (1.04, 3.49)**	318/1451 (21.9)	1.59 (0.75, 3.36)	1428/4504 (31.7)^*^	**1.72 (1.18, 2.51)**
Support from grandmothers ^b^								
No	15/89 (16.9)	1.00 (reference)	30/95 (31.6)	1.00 (reference)	22/98 (22.4)	1.00 (reference)	67/282 (23.8)	1.00 (reference)
Yes	560/1592 (35.2)^*^	1.22 (0.51, 2.91)	566/1545 (36.6)	0.75 (0.39, 1.46)	321/1477 (21.7)	0.65 (0.32, 1.33)	1447/4614 (31.4)^*^	0.77 (0.51, 1.17)
Support from the best friends ^b^								
No	23/129 (17.8)	1.00 (reference)	42/133 (31.6)	1.00 (reference)	21/139 (15.1)	1.00 (reference)	86/401 (21.4)	1.00 (reference)
Yes	539/1498 (36.0)^*^	**2.40 (1.28, 4.50)**	532/1422 (37.4)	1.33 (0.82, 2.16)	307/1380 (22.2)	1.51 (0.80, 2.82)	1378/4300 (32.0)^*^	**1.64 (1.19, 2.26)**
Provision of breastfeeding education by hospitals								
No	127/384 (33.1)	1.00 (reference)	115/376 (30.6)	1.00 (reference)	50/342 (14.6)	1.00 (reference)	292/1102 (26.5)	1.00 (reference)
Yes	460/1317 (34.9)	0.98 (0.74, 1.30)	491/1279 (38.4)^*^	1.21 (0.92, 1.61)	301/1251 (24.1)^*^	1.43 (0.99, 2.06)	1252/3847 (32.5)^*^	1.14 (0.96, 1.35)
Employment and paid maternity leave (PML) ^a^								
Unemployment	194/595 (32.6)	1.00 (reference)	186/562 (33.1)	1.00 (reference)	118/567 (20.8)	1.00 (reference)	498/1724 (28.9)	1.00 (reference)
Informal employment	197/560 (35.2)	0.99 (0.76, 1.29)	189/568 (33.3)	1.13 (0.86, 1.49)	92/534 (17.2)	0.88 (0.63, 1.22)	478/1662 (28.8)	1.00 (0.85, 1.18)
Formal employment								
without PML	29/83 (34.9)	0.92 (0.53, 1.57)	30/78 (38.5)	1.38 (0.82, 2.31)	13/78 (16.7)	0.88 (0.44, 1.76)	72/239 (30.1)	1.03 (0.75, 1.42)
with 0–2 months of PML	5/28 (17.9)	0.42 (0.14, 1.19)	20/34 (58.8)	**3.09 (1.43, 6.69)**	4/20 (20.0)	0.95 (0.30, 3.03)	29/82 (35.4)	1.24 (0.75, 2.04)
with 3–5 months of PML	154/411 (37.5)	1.00 (0.70, 1.43)	166/390 (42.6)	1.33 (0.93, 1.90)	108/366 (29.5)	**1.68 (1.12, 2.53)**	428/1167 (36.7)	1.22 (0.99, 1.51)
with ≥6 months of PML	8/24 (33.3)	1.02 (0.38, 2.76)	14/22 (63.6)^*^	**3.27 (1.27, 8.37)**	16/27 (59.3)^*^	**6.74 (2.81, 16.16)**	38/73 (52.1)^*^	**2.77 (1.65, 4.65)**
Social support ^a^								
No	473/1437 (32.9)	1.00 (reference)	506/1422 (35.6)	1.00 (reference)	297/1358 (21.9)	1.00 (reference)	1276/4217 (30.3)	1.00 (reference)
Yes	109/257 (42.4)^*^	**1.64 (1.22, 2.21)**	98/228 (43.0)^*^	**1.63 (1.19, 2.24)**	54/232 (23.3)	1.18 (0.81, 1.71)	261/717 (36.4)^*^	**1.49 (1.24, 1.78)**

EBF indicates exclusively breastfed infants, BF indicates breastfed infants, and aORs indicates adjusted odds ratios

aOR were adjusted for residents, infant sex and age of months, and other variables listed in the table

^*^ *p* value < 0.05 for univariate analysis

^a^ Listwise deletion in logistic regression was used to handle missing data of these variables with < 1% missing data

^b^ A category “unknown” was assigned to the missing value of these variables with ≥1% missing data; these cases were also included in the analysis but the results are not presented

Subgroup analysis shows that these associations varied across age groups (Table 2). The association of caesarean delivery with low probability of exclusive breastfeeding was observed in breasted infants in all three age groups according to the univariate analysis or multivariable adjusted analyses, while the significant association between infant disease history and low probability of exclusive breastfeeding was observed only in breasted infants aged 0–3 months. Results of the univariate analysis showed that higher maternal education was associated with increased exclusive breastfeeding proportion in breastfed infants of all age groups, but multivariable adjusted analyses found that aORs decreased with increased infant age and was only statistically significant for the association of maternal education level of college school or higher (aOR 1.44; 95% CI 1.03, 2.01) with exclusive breastfeeding in breastfed infants aged 0–1 month. Mothers had support for breastfeeding from their husbands (aOR 1.91; 95% CI 1.04, 3.49) and best friends (aOR 2.40; 95% CI 1.28, 4.50) were more likely to exclusively breastfeed their infants compared to those without such support, which was statistically significant among breastfed infants aged 2–3 and 0–1 month, respectively. No significant association was found between maternal employment status and exclusive breastfeeding in breastfed infants aged 0–1 month. In breastfed infants aged 2–3 months and 4–5 months, proportion of exclusive breastfeeding was higher in those whose mothers were formally employed and with longer paid maternity leave compared to those of unemployed mothers. Significant association between social support for exclusive breastfeeding was found in breastfed infants aged 0–1 month (aOR 1.64; 95% CI 1.22, 2.21) and 2–3 months (aOR 1.63; 95% CI 1.19, 2.24).

The associations of maternal breastfeeding experience and knowledge with exclusive breastfeeding among breastfed infants are presented in Table 3. Compared with mothers feeding their first child, mothers never or ever breastfeeding a child for less 6 months were less likely to exclusively breastfeed the infants (aOR 0.61; 95% CI 0.44, 0.84), and those ever breastfeeding a child for ≥6 months (aOR 1.29; 95% CI 1.10, 1.51) and those with early initiation of breastfeeding (aOR 1.36; 95% CI 1.13, 1.64) were more likely to exclusively breastfeed the infants. Knowing the perception of exclusive breastfeeding (aOR 1.61; 95% CI 1.40, 1.84), and having higher scores on the knowledge of benefits of breastfeeding (aOR 1.38; 95% CI 1.18,1.62) were associated with higher probability of exclusive breastfeeding; Subgroup analysis showed that the associations were also significant in breastfed infants aged 2–3 months and 4–5 months, and even in those aged 0–1 month for knowing the perception of exclusive breastfeeding.

**Table 3:** The association of maternal experiences and knowledge with exclusive breastfeeding among breastfed infants under 6 months

	0–1 months		2–3 months		4–5 months		Total	
	EBF/BF (%)	aOR (95%CI)	EBF/BF (%)	aOR (95%CI)	EBF/BF (%)	aOR (95%CI)	EBF/BF (%)	aOR (95%CI)
		(*n* = 1654)		(*n* = 1621)		(*n* = 1553)		(*n* = 4828)
Ever breastfed a child ^a^								
First birth	289/819 (35.3)	1.00 (reference)	274/764 (35.9)	1.00 (reference)	161/712 (22.6)	1.00 (reference)	724/2295 (31.5)	1.00 (reference)
Never or for < 6 months	16/94 (17.0)	**0.46 (0.25, 0.83)**	26/111 (23.4)	0.74 (0.44, 1.22)	14/83 (16.9)	0.73 (0.38, 1.39)	56/288 (19.4)	**0.61 (0.44, 0.84)**
For ≥6 months	278/779 (35.7)^*^	1.22 (0.94, 1.59)	304/773 (39.3)^*^	**1.58 (1.22, 2.05)**	171/791 (21.6)	1.00 (0.73, 1.37)	753/2343 (32.1)^*^	**1.29 (1.10, 1.51)**
Early initiation of breastfeeding ^b^								
No	481/1449 (33.2)	1.00 (reference)	501/1428 (35.1)	1.00 (reference)	293/1371 (21.4)	1.00 (reference)	1275/4248 (30.0)	1.00 (reference)
Yes	100/231 (43.3)^*^	1.34 (0.98, 1.82)	93/201 (46.3)^*^	**1.46 (1.06, 2.02)**	53/194 (27.3)	1.27 (0.87, 1.84)	246/626 (39.3)^*^	**1.36 (1.13, 1.64)**
Knowing about exclusive breastfeeding								
No	213/728 (29.3)	1.00 (reference)	209/689 (30.3)	1.00 (reference)	89/642 (13.9)	1.00 (reference)	511/2059 (24.8)	1.00 (reference)
Yes	374/973 (38.4)^*^	**1.50 (1.19, 1.88)**	397/966 (41.1)^*^	**1.55 (1.23, 1.94)**	262/951 (27.5)^*^	**2.12 (1.59, 2.83)**	1033/2890 (35.7)^*^	**1.61 (1.40, 1.84)**
Score on the knowledge of benefits of breastfeeding ^a^								
< 8	437/1297 (33.7)	1.00 (reference)	396/1229 (32.2)	1.00 (reference)	236/1198 (19.7)	1.00 (reference)	1069/3724 (28.7)	1.00 (reference)
≥ 8	148/398 (37.2)^*^	1.09 (0.83, 1.44)	208/422 (49.3)^*^	**1.72 (1.32, 2.23)**	113/388 (29.1)^*^	**1.53 (1.12, 2.10)**	469/1208 (38.8)^*^	**1.38 (1.18, 1.62)**

EBF indicates exclusively breastfed infants, BF indicates breastfed infants, and aORs indicates adjusted odds ratios

aORs were adjusted for residents, infant sex and age of months, and factors identified to be significantly associated with EBF in Table 2

^*^ *p* value < 0.05 for univariate analysis

^a^ Listwise deletion in logistic regression was used to handle missing data of these variables with < 1% missing data

^b^ A category “unknown” was assigned to the missing value of these variables with ≥1% missing data; these cases were also included in the analysis but the results are not presented

## Discussion

### Current situation of exclusive breastfeeding in China

Our results show that 29.5% of infants under 6 months were exclusively breastfed in China as the measurement of 24-h food recall. The rate is higher by 10 percentage points than that estimated in 2013 [7], but still lower than that in other low- and middle-income countries [4], with the same food recall method. Worldwide, 43 of the 188 countries with available data have reached the WHA target of ≥50% exclusive breastfeeding rate by 2025, as measured by 24-h food recall, and even in Asia, several countries including Bangladesh, Laos and Myanmar, have achieved rapid growth in exclusive breastfeeding rates [20]. Scaling up exclusive breastfeeding remains an exacting public health work for China. Our findings may contribute to improve breastfeeding programs by expanding the knowledge about the determinants of exclusive breastfeeding.

### The importance of breastfeeding knowledge

We found that a relatively large reduction in exclusive breastfeeding and increased initiation of semi-solid/solid foods began in the fifth month of infancy. Infants at this age were at risk for early-onset undernutrition such as anemia, which may result from the insufficiency of breast milk to meet the nutritional needs of infants [21–23]. No or insufficient breast milk is the foremost reason given for non-commencement or early cessation of breastfeeding by almost two thirds of participants who had stopped breastfeeding in our study. However, previous studies indicate that inadequate or incorrect knowledge of breastfeeding may also misguide mothers to think breast milk insufficient [17, 24]. For a long time in the past, Chinese mothers used to introduce complementary foods when their babies were 4–6 months old, which was even recommended in the feeding guidelines published in the first decade of the twenty-first century [25]. Therefore, it is important to provide breastfeeding education and counselling to improve maternal breastfeeding practices [24, 26]. Our findings also suggest that good breastfeeding knowledge was associated with an increase in exclusive breastfeeding across all age groups under 6 months. However, unfortunately, we didn’t identify the significant impact of breastfeeding education of health institutions, which deserves rethink about the program strategy and effectiveness.

### Benefits of maternal and infant health for the sustenance of breastfeeding

Consistent with previous studies [11, 27], this study suggests the benefits of health of mother and infant for the sustenance of breastfeeding, especially in the first 4 months after birth. Maternal and child illness can cause an interruption of breastfeeding and decrease in lactation, and then contribute to early breastfeeding cessation or failure of breastfeeding initiation [12, 17, 28]. Our results also strengthen the inference that caesarean delivery had a significant detrimental effect on early and exclusive breastfeeding [29]. Women who give birth by caesarean section usually experience a longer elapsed time between birth and putting their baby to the breast than women who labored vaginally [30]. Early separation especially in the first postnatal hours has strong and harmful effects on the duration and success of breastfeeding [31]. Delayed onset of lactation, disrupted mother-infant interaction and inhibited infant suckling may mediate the effects of caesarean delivery on breastfeeding [29]. In China, about one third live births is caesarean and the rate is still likely to increase in the coming years [32]. How to reduce caesarean section and its adverse effect on breastfeeding is a serious public health problem unsolved.

### Family, peer, workplace and sociocultural support for breastfeeding

This study further deepens our understanding of the association of familial, peer, and workplace factors with maternal breastfeeding intention, which varied across different age of infants. In breastfed infants aged 2–5 months, those whose mothers were formally employed and received relatively long duration of paid maternity leave, were more likely to be exclusively breastfed than those of unemployed mothers. Such significant association was not found in those aged 0–1 month. These findings indicate the opportunity cost of exclusive breastfeeding for mothers and their choices between working for money and continuing to exclusively breastfeed the child. Maternal employment was identified as a protective factor for exclusive breastfeeding in America and Ghana [14, 33], but a risk factor in other developing countries [14, 27, 34]. This difference may be caused by different social security and welfare of the employers in the survey areas. Surprisingly, our study showed that support of husbands and peers, but not that of grandmothers, was significantly associated with increased exclusive breastfeeding. This finding is supported by an intervention study conducted by Min Su and Yanqiong Ouyang [35]. In their study, encouraging fathers to give mothers more physical and emotional support was more likely to improve the success and duration of exclusive breastfeeding than only providing breastfeeding education for mothers.

Our findings also reveal that social and cultural traditions have a profound impact on feeding beliefs and behaviors in China. The stigma of viewing breastfeeding by strangers haunts most of Chinese women and their partners. In our study, only 15% of mothers reported their public breastfeeding decisions were not limited by social attitudes. A study shows even in those highly educated population such as undergraduate students, more than half of them were unwilling to breastfeed or accept their partner’s breastfeeding in public [36]. Similar awkwardness of public breastfeeding exists in many countries [15, 37, 38]. Women who breastfeed in public were viewed as lacking self-respect. Grant argued in her research that this view originates in unequal gender relationships in society and the framing of breasts as sexual rather than nurturing [37]. Reversing this perception to reduce the negative impact on breastfeeding will be a very difficult and time-consuming task.

### Potential implications

China has a quite low rate of exclusive breastfeeding despite many years of efforts to increase it. Along with the many positive health effects that breastfeeding confers, more effective policies and programs to increase optimal breastfeeding could result in substantial public health gains. Our findings highlight a need to develop a comprehensive intervention framework to support children, mothers, as well as their families. Group lessons on breastfeeding is also recommended according to our finding about the association between the best friends’ support and exclusive breastfeeding. Health institutions need take actions to reduce caesarean section and improve maternal and child health services. Further efforts are also warranted to improve social and policy support for childbearing women at the population level so as to increase breastfeeding duration and exclusivity, including improving social and cultural acceptance of public breastfeeding, providing special rooms for breastfeeding in public places, guaranteeing the equity and welfare of women’s employment, giving enough paid maternity leave for mothers and building a breastfeeding-friendly workplace.

### Limitations of the study

This study has some limitations. First, the survey only enrolled infants who were primarily cared for by their mothers, which can lead to overestimation of the exclusive breastfeeding rate and bias in the results of risk factor analyses. Second, although this study covered many potential breastfeeding determinants of multiple levels, there are still some important factors that failed to be measured, such as maternal mental health. Some variables, such as household income, are not included in the analysis because of too much missing data. Third, natural shortcomings of cross-sectional design exist in this study, and the causal relationships discussed are still needs further validation by prospective observational studies.

## Conclusions

In summary, although great improvement has been made in the past 5 years, the exclusive breastfeeding rate is still very low in China. We found that about one third of infants under 6 months were exclusively breastfed in China. None or insufficient breast milk is the foremost reason given for non-commencement or early cessation of non-breastfeeding by almost two thirds of participants who had stopped breastfeeding. Among breastfed infants, individual factors, including caesarean delivery, infant disease history, and maternal low education and age of ≥40 years, were associated with decreased exclusive breastfeeding, while setting and sociocultural factors including being formally employed with relatively long paid maternity leave, husbands and best friends’ support for breastfeeding, and supportive environment for breastfeeding in public, were associated with increased exclusive breastfeeding. A comprehensive intervention framework is needed to increase optimal breastfeeding and achieve substantial public health gains. Future research needs to continuously monitor the trend of exclusive breastfeeding rate and explore effective intervention strategies.

## Data Availability

The questionnaire and datasets used during the current study are available from the corresponding author on reasonable request.

## Abbreviations

CI: Confidential interval
OR: Odds ratio
PML: Paid maternity leave
SD: Standard deviation
WHA: World health assembly
WHO: World health organization
